# 1,4-*β*-d-Glucomannan from *Dendrobium officinale* Activates NF-*к*B via TLR4 to Regulate the Immune Response

**DOI:** 10.3390/molecules23102658

**Published:** 2018-10-16

**Authors:** Yan-Ping Huang, Tao-Bin He, Xian-Dan Cuan, Xuan-Jun Wang, Jiang-Miao Hu, Jun Sheng

**Affiliations:** 1 Key Laboratory of Pu-er Tea Science, Ministry of Education, Yunnan Agricultural University, Kunming 650201, China; fengzhongjiaren00@163.com (Y.-P.H.); hetaobin@mail.kib.ac.cn (T.-B.H.); cuanxiangdan@163.com (X.-D.C.); 2 College of Science, Yunnan Agricultural University, Kunming 650201, China; 3 College of Food Science and Technology, Yunnan Agricultural University, Kunming 650201, China; 4 Kunming Institute of Botany, Chinese Academy of Sciences, Kunming 650201, China

**Keywords:** *Dendrobium officinale*, 1,4-*β*-d-glucomannan, thermal stability, NF-*к*B, TLR4

## Abstract

2,3-*O*-acetylated-1,4-*β*-d-glucomannan (DOP-1-1) is a polysaccharide isolated from the stem of *Dendrobium officinale*. DOP-1-1 has been demonstrated to have remarkable immunomodulatory properties, but little is known about the influence of its structural diversity on bioactivity (and even less about the exact mechanism underlying its immune responses). First, DOP-1-1 was stabilized at different temperatures and pH conditions based on differential scanning calorimetry and size exclusion-chromatography–high-performance liquid chromatography. Then, a detailed study on the effects of DOP-1-1 on a human leukemia monocytic cell line (THP-1) under normal conditions was undertaken. DOP-1-1 promoted the translocation of nuclear factor-kappa B (NF-*κ*B) and degradation of I*κ*B proteins. The expression of genes and proteins closely associated with the immune, survival and apoptotic functions of NF-*κ*B were analyzed by quantitative real-time RT-PCR. Furthermore, CCL4 and IP10 were confirmed to be the novel targets of the immune response stimulated by DOP-1-1. The phosphorylation of NF-*к*B was inhibited by treatment with a toll-like receptor 4 (TLR4) antagonist (TAK-242) and myeloid differentiation factor 88 (MyD88) inhibitor (ST2825). These data suggested: (i) the *O*-acetylated glucomannan DOP-1-1 is present in the steady state in low-pH solutions; (ii) DOP-1-1 can induce an immune response through NF-*к*B mediated by a TLR4 signaling pathway; and (iii) CCL4 and IP10 could be the novel targets of the immune response stimulated by *O*-acetylated glucomannan.

## 1. Introduction

Dendrobii Officinalis Caulis, the stems of *Dendrobium officinale* Kimura et Migo, is a Chinese herbal medicine and functional food. It has been used for decades because it has immunomodulatory [1] antineoplastic [2], antioxidative [3] and antifungal activities [4]. It is also used in combination with other tonic Chinese medicines, such as “Panacis Quinquefolii Radix” (American Ginseng), “Lycii Fructus” (Barbary Wolfberry Fruit), and “Dioscoreae Rhizoma” (Rhizome of Common Yam).

In addition, polysaccharides, as the major active constituent of *Dendrobium* species, recently have been reported to have immunomodulatory, antioxidant, and antitumor effects, to inhibit apoptosis, and to prevent liver injury and fibrosis [5]. Moreover, the conformational changes of biopolymers, such as proteins, lipids, and polysaccharides have pivotal roles in biologic activities [6,7,8,9,10]. However, the study about the relationship between the conformational dynamics of polysaccharides and biological processes are scarce.

Polysaccharides are not only basic nutrients but also play a key part in herbal medicine [11]. Additionally, it is commonly believed that polysaccharides have immunoregulatory activities [12,13,14]. However, the immune responses of various polysaccharides refer to different signaling pathways [15,16].

The regulation of immunity plays an important part in life processes and the treatment of diseases [17,18]. The immune system of an organism provides an extraordinary defense against infections, where innate immunity is the first line of defense in mediating the initial protection against foreign attacks. Once it recognizes matter as “non-self”, it activates multiple chemical and physiologic processes to control and eliminate the pathogen. Thus, the immune response is associated with driving signaling pathways such as nuclear factor-kappa B (NF-κB) [19] and janus kinase/signal transducers and activators of transcription (JAK/STAT) [20,21].

**Figure 1:**
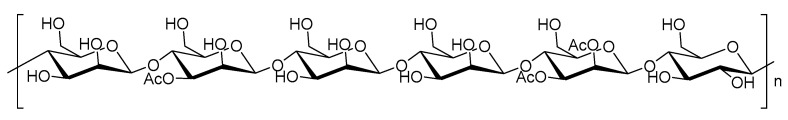
The structure of *O*-acetylated glucomannan according to published papers [22,24,25].

The immunomodulatory mechanism of action of the polysaccharide *O*-acetylated glucomannan from *D. officinale* has not been studied even though its immunomodulatory activities have been noted widely [1,22,23]. In our previous study, *O*-acetylated glucomannan from *D. officinale* showed remarkable immune-enhancing properties [22]. Additionally, the thermal stability of polysaccharides from *D. officinale* under a thermal transition temperature with changing pH has not been reported although its structural characterization has been fully elucidated [24,25]. In fact, *O*-acetylated glucomannan was shown to have a molecular weight of 1.78 × 10^5^ Da and a backbone composed of →4)-Manp-(1→ and →4)-Glcp-(1→ residues (Figure 1).

Taking the thermal stability of *O*-acetylated glucomannan into consideration, the immunomodulatory mechanism of action of *O*-acetylated glucomannan was further investigated under normal conditions. This study was carried out because *O*-acetylated glucomannan from *D. officinale* is widely used in ethnopharmacology and the functional-food industry.

## 2. Results

### 2.1. Carbohydrate and Protein Contents of Crude and Pure O-Acetylated Glucomannan

In our previous study, the DOP-1-1 (a type of *O*-acetylated glucomannan) present in *D. officinale* was isolated by HPLC [22]. To further confirm the homogeneity of *O*-acetylated glucomannan isolated from *D. officinale*, carbohydrate and protein contents were measured.

The total sugar content of *D. officinale* increased from 76% (*w/w*, DOP, after dialysis) to 99% (*w/w*, DOP-1-1) after purification by HPLC [22], which explains the decrease in the percentage content of contaminating proteins to zero after purification (Table 1).

### 2.2. Stability of DOP-1-1 under Different pH Conditions

Biopolymers are induced by intramolecular non-covalent forces to fold into well-defined tertiary structures [26]. Therefore, DSC was used to ascertain the thermal stability of DOP-1-1 under different pH conditions.

**Figure 2:**
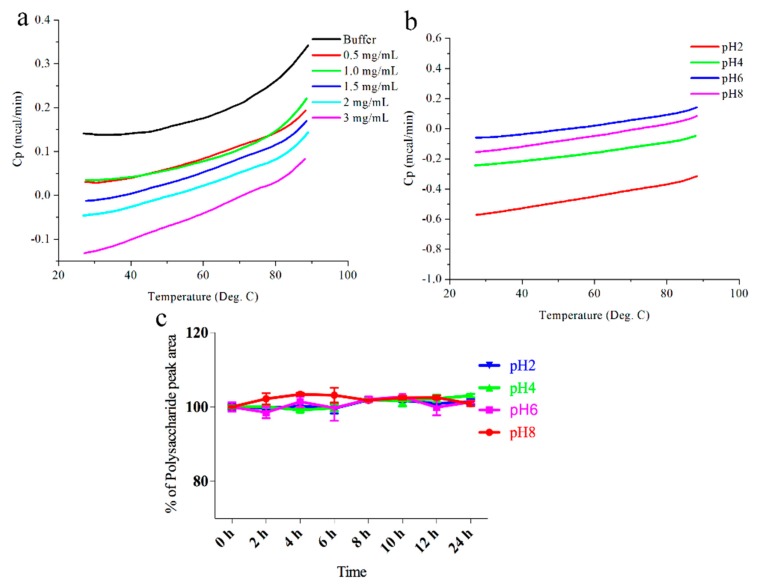
The stability of DOP-1-1. (**a**) DSC scans of different concentrations of DOP-1-1 dissolved in water. (**b**) DSC scans of DOP-1-1 (2 mg/mL) at pH 2, pH 4, pH 6, and pH 8 solution. (**c**) Stability of DOP-1-1 (2 mg/mL) at 60 °C. Comparison of DOP-1-1 peak percentage at different time points for pH 2, pH 4, pH 6, and pH 8 solution. All percentage values of HPLC data are normalized against the values at 0 h.

Little change in DOP-1-1 was observed in different pH solutions (Figure 2b) compared with buffer, which may have been due to unfolding or a monomeric state of DOP-1-1. Thus, the DSC profiles of different concentrations of DOP-1-1 were further analyzed. Similarly, little change was observed among these samples (Figure 2a,b). These results suggested that DOP-1-1 might be extremely stable under different pH conditions (Figure 2b). To further confirm this conclusion, the stability of DOP-1-1 under different pH conditions was examined with HPLC. HPLC data also demonstrated that DOP-1-1 was insensitive to pH (Figure 2c). There are many challenges to overcome when developing manufacturing processes for polysaccharides, such as temperature and pH.

### 2.3. Toxicity of DOP-1-1 to THP-1 Monocytes

**Figure 3:**
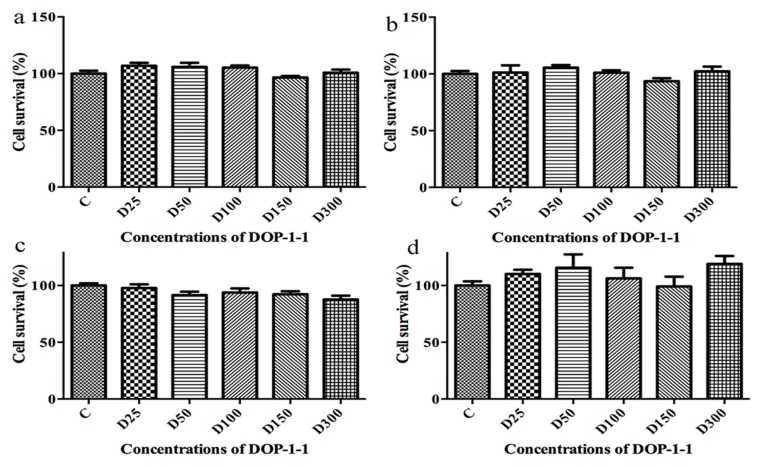
Effects of DOP-1-1 at different concentrations on the survival of mature macrophages differentiated from THP-1 cells by MTT assay. (**a**) 12 h, (**b**) 24 h, (**c**) 36 h, (**d**) 48 h (D25, D50, D100, D150, D300 means 25, 50, 100, 150, 300 μg/mL of DOP-1-1. The data were expressed as mean values ± SD).

THP-1 is a human leukemia monocytic cell line that has become a common model to estimate the modulation of monocyte and macrophage activities. As THP-1 monocytes are a type of terminally differentiated cells [27], the toxicity of DOP-1-1 to mature macrophages was examined. Results suggested that DOP-1-1 had absolutely no effect on macrophages derived from a human leukemia monocytic cell line (Figure 3a–d).

### 2.4. Effects of DOP-1-1 on NF-κB Signaling

**Figure 4:**
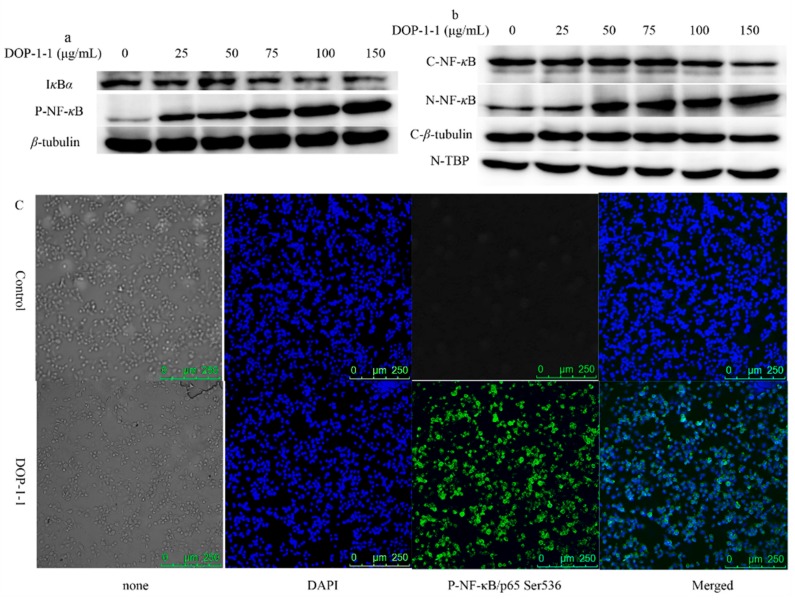
DOP-1-1 mediated immune response via NF-*κ*B. (**a**) Western blot of phosphorylated NF-*κ*B and I*κ*B*α* from whole proteins. (**b**) Western blot for cytoplasmic and nuclear fraction: C-NF-*κ*B and N-NF-*κ*B represent NF-*κ*B proteins in cytoplasm and nucleus, respectively; C-*β*-tubulin and N-TBP (TATA binding protein) were the internal reference for cytoplasm and nucleus, respectively. (**c**) Immunofluorescence of phosphorylated NF-*κ*B.

Our previous study showed that DOP-1-1 has immunomodulatory properties [22]. To further study the mechanism of DOP-1-1-induced immunoregulation, the proteins associated with immune responses were examined. The cellular response to different concentrations of DOP-1-1 resulted in a phosphorylation increase of NF-*κ*B in a dose-dependent manner and degradation of I*κ*B*α* (Figure 4a). Other proteins were not detected. NF-*κ*B pathway can be activated via at least three distinct routes: Canonical, atypical and noncanonical pathways. Our results suggested that DOP-1-1 induced activation of NF-*κ*B signaling via I*κκ*- independent means (I*κκ*-*β* and I*κκ*-*α* were not detected). The activation of NF-κB can induce its nuclear translocation. In order to ascertain whether DOP-1-1 translocated NF-*κ*B into the nucleus, western blotting for cytoplasmic and nuclear fractions was carried out using a Nuclear and Cytoplasmic Protein Extraction Kit. The expression of NF-*κ*B (p65) in the cytoplasmic fractions of THP-1 cells decreased, and that of NF-*κ*B (p65) in nuclear extracts increased, in a dose-dependent manner (Figure 4b). In order to confirm these results, NF-*κ*B expression was determined by an immunofluorescence method using a secondary antibody (Alexa Fluor 488). Green fluorescent staining was stronger in the nucleus than in plasma (Figure 4c), a result that was consistent with the data of using a Nuclear and Cytoplasmic Protein Extraction Kit. Taken together, these results demonstrated that DOP-1-1 induced an immune response via the NF-*κ*B pathway.

### 2.5. TLR4 Is a Pattern-Recongnition Receptor for DOP-1-1

**Figure 5:**
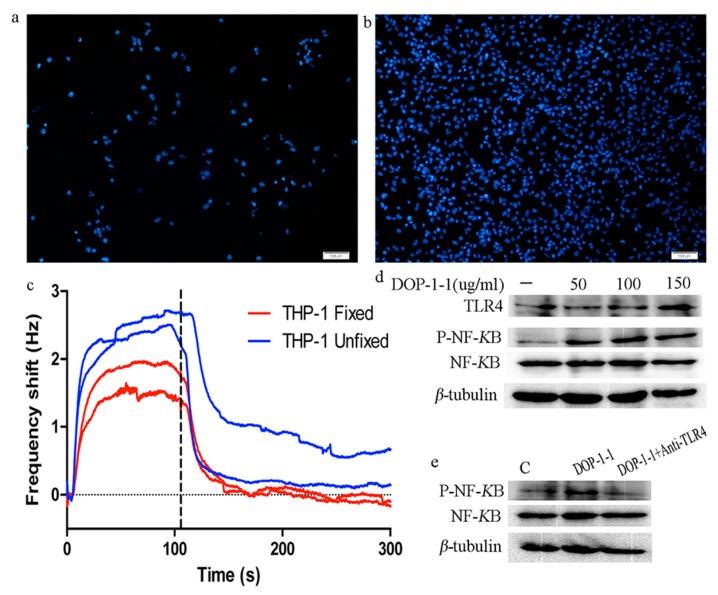
Interactions between DOP-1-1 and protein receptors on THP-1 cells. (**a**,**b**) Evaluation of Cell coverage on sensor surface test: QCM measurements by staining the nuclei of THP-1 cells with Hoechst33342 and visualized under a fluorescent microscope. (**c**) Sensograms and binding data for each DOP-1-1 and THP-1 cells. (**d**) Effects of DOP-1-1 on TLR4 and NF-*κ*B protein and *β*-tubulin. (**e**) Effects of DOP-1-1 on NF-*κ*B protein by blocking TLR4 and *β*-tubulin.

In order to detect the specificity and affinity of DOP-1-1 to the surface receptors on THP-1 cells, the latter were grown on COP-1 chips (compatible polystyrene coated sensor surface). A large shift in the frequency was observed and most of the cells were washed away during the analysis (Figure 5a). This observation suggested that cells did not adhere strongly to the surface of THP-1 cells. Hence, the protocol was changed and COP-1 chips were coated with poly-*L*-lysine. Most cells remained on the sensor surface by fixation, whereas without fixation the THP-1 cells were washed away by the flow (Figure 5b). However, a similar low-frequency shift was detected in fixed and unfixed cells (Figure 5c). Combining these results, we could conclude that DOP-1-1 had very little interaction with the receptors on THP-1cells.

TLR4 has a central role in the enhancement of the innate immune response and the production of cytokines induced by polysaccharides. Therefore, TLR4 expression was analyzed by western blotting. The expression of TLR4 protein increased in a dose-dependent manner (Figure 5d). In order to ascertain whether TLR4 is a pattern-recognition receptor for DOP-1-1, blockade of TLR4 protein using anti-mouse TLR4 monoclonal antibodies was employed to interfere in the interaction between DOP-1-1 and TLR4. Upon addition of anti-mouse TLR4 monoclonal antibodies, the DOP-1-1-induced NF-*κ*B activation in THP-1 cells was strongly inhibited (Figure 5e), which suggested that DOP-1-1 stimulated the immune response via TLR4.

### 2.6. Expression of the Genes Associated with Nf-Κb Signaling

To further test the mechanism of NF-*κ*B activation and targets of the immune response induced by DOP-1-1, the expression of some of the genes involved in the immune response was measured by treatment with PDTC and Bay 11-7082.

**Figure 6:**
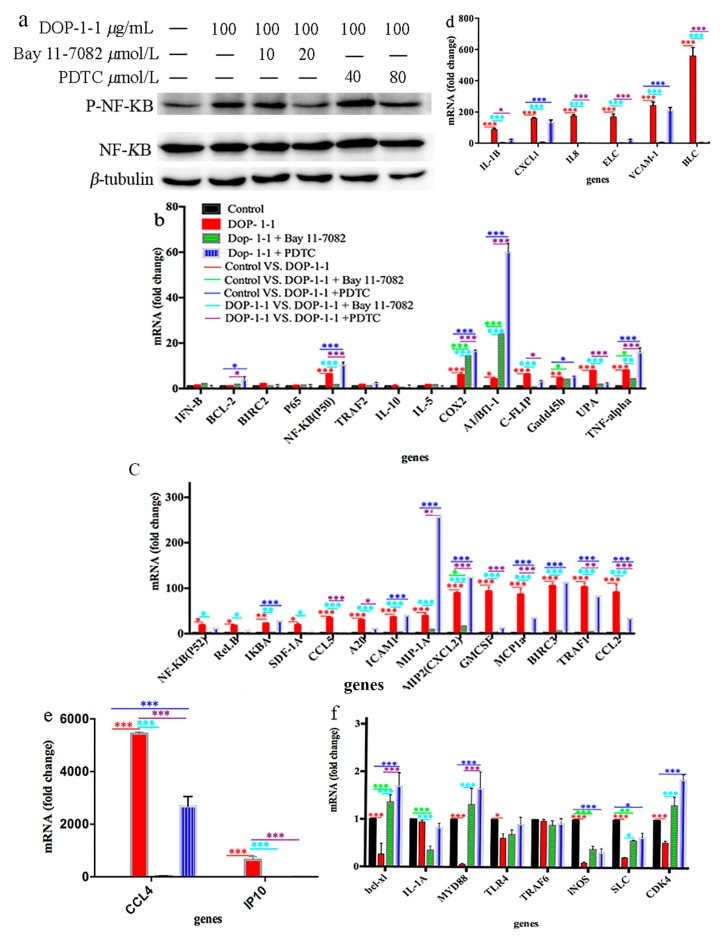
Comparison of the expression profile of genes associated with NF-*κ*B between DOP-1-1treated cells and untreated cells. (**a**) The effects of PDTC and bay 11-7082 (NF-*κ*B inhibitor) on NF-*κ*B (**b**) mRNA fold changing ranged from 1 to 10; (**c**) mRNA fold changing ranged from 10 to 150; (**d**) mRNA fold changing ranged from 150 to 1000; (**e**) mRNA fold changing ranged from 2000 to 6000. (**f**) DOP-1-1 down-regulated genes; (Each assay was performed in triplicate. * *p* < 0.05, ** *p* < 0.01, *** *p* < 0.001).

PDTC (80 μmol/L) and Bay 11-7082 (20 μmol/L) inhibited the phosphorylation of NF-*κ*B (Figure 6a). RT-PCR analyses suggested that DOP-1-1 gave rise to changes in the expression of almost all of the genes associated with NF-*κ*B (including those associated with inflammation, survival, and apoptosis) compared with control (Figure 6b–f) but pretreatment with PDTC (80 μmol/L) or Bay 11-7082 (20 μmol/L) resulted in reduced expression of many of these genes. These results provided further evidence that DOP-1-1 induced an immune response through the NF-*κ*B pathway. Besides, expression of CCL4 and IP10 showed the greatest changes among these genes (Figure 6 and Table 2, 4023- and 2954-fold higher, respectively). Further, the up-regulation of the expression of CCL4 and IP10 could be suppressed after treatment with the NF-*κ*B inhibitors PDTC and Bay 11-7082, which confirmed that CCL4 and IP10 had a specific response to the NF-*κ*B activation induced by DOP-1-1. Therefore, CCL4 and IP10 could be considered to have molecular signatures in the immunoregulation by DOP-1-1.

## 3. Discussion

The water extracts of *D. officinale* can be infused in tea or wine, or consumed as a decoction, by sick individuals. Polysaccharides, as the major active component of *D. officinale*, encompass many physicochemical, and physiologic effects that could stimulate the immune system.

In the present study, the conformational change of DOP-1-1 was analyzed. Our results showed that DOP-1-1 had good stability under pH ranged from 2 to 8(Figure 2a–c). So, in order to fully extracted the polysaccharide from *D. officinale*, a small amount of an acid or base may be added in solvent. This observation is in contrast with researchers who have often assumed that polysaccharides in acidic solutions can be readily degraded. This assumption may be due to the DOP-1-1 linkage type, which is composed of →4)-Manp-(1→ and →4)-Glcp-(1→ residues (Figure 1) [22,24,25].

In jawed vertebrates, the immune response is composed of innate immunity and adaptive immunity. The former is carried mainly by macrophages and neutrophils, which are the first line of defense against infections. The innate response is usually triggered when microbes are identified by pattern-recognition receptors (PRRs). But, as polysaccharides cannot enter cells directly because of their large molecular mass, the first step for polysaccharides to exert their functions is the recognition by PRRs [28,29].

This study revealed that DOP-1-1 was weakly bound to the surface of THP-1 cells (Figure 5a–c). There are two explanations for this result. First, very few receptors may be expressed on the surface of THP-1 cells. Second, the number of receptors may be sufficient, but there might be weak interactions between DOP-1-1 and the proteins on the surface of THP-1 cells [30]. In addition, different polysaccharides can be recognized by different PRRs, such as TLR2–4, CR-3, and NOD-like receptor protein (NLRP)1–7 [12,31]. Among these receptors, TLR4 has been reported to be the most common PRR for the recognition of natural polysaccharides [32]. In order to ascertain whether TLR4 is a PRR of DOP-1-1 when it binds to macrophages, the changes of TLR4 expression was analyzed by treatment with DOP-1-1 (Figure 5d). Further, previous treatment with an anti-TLR4 antibody inhibited NF-*к*B activation (Figure 5d), which suggested that DOP-1-1 activates macrophages via TLR4-mediated signaling pathways.

It would be useful to understand the recognition pattern of *O*-acetylated glucomannan. Hence, the pathways associated with the immune response were systematically investigated. DOP-1-1 promoted the degradation of I*к*B complexes and activated NF-*к*B phosphorylation (Figure 4a). Once activated, the NF-*к*B dimer translocates to the nucleus, where it binds to specific DNA sequences [33]. Therefore, the changes in levels of NF-*к*B proteins in the cytoplasm and nuclei were also tested. Finally, the expression of proteins and genes associated with the NF-*к*B signaling pathway were tested by RT-PCR in this study. When inhibitors of NF-*к*B phosphorylation were added, the gene-expression levels were similar to those of control. Taken together, our results showed that *O*-acetylated glucomannan induced an immune response through NF-*к*B mediated by a TLR4 signaling pathway. Furthermore, CCL4 and IP10 were confirmed to be the novel targets of the immune response stimulated by *O*-acetylated glucomannan.

CCL4 and IP10 are chemotactic cytokines that have a vital role in the regulation of migration of various body cells, especially immune cells such as monocytes, macrophages, neutrophils, natural killer cells and T cells. The importance of chemokines has been shown in recent years. It has become recognized that they are key players in many disease processes, including inflammation, autoimmune disease, infectious diseases, and cancer [34,35]. Our data suggested that DOP-1-1 upregulated expression of CCL4 and IP10, which could provide new targets for the development of immunologic intervention strategies. Our results also suggest that DOP-1-1 could have an important role in the treatment of autoimmune disease, infectious diseases, wound healing, and cancer.

**Figure 7:**
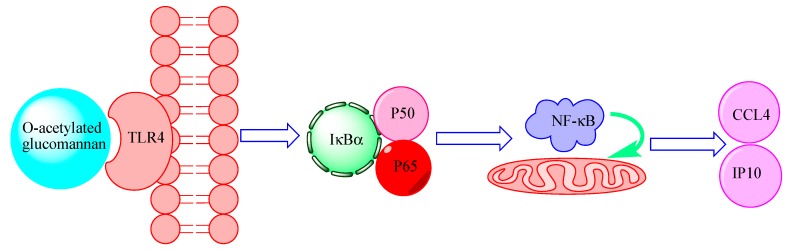
Schematic diagram depicting the immunomodulatory mechanism of *O*-acetylated glucomannan.

The immunoregulatory mechanisms of polysaccharides vary depending on the type of in polysaccharide [12,13,14,36]. A hypothesis for the immunoregulatory mechanism of DOP-1-1 is shown in Figure 7.

## 4. Materials and Methods

### 4.1. Materials and Reagents

*O*-acetylated glucomannan in the form of 2,3-*O*-acetylated-1,4-*β*-d-glucomannan (DOP-1-1) was isolated from the stems of *D. officinale* collected in Pu-er city in Yunnan Province (China) in September 2013 [22]. Phenylmethylsulfonyl fluoride (PMSF), phorbol-12-myristate-13-acetate (PMA), and 3-(4,5-dimethyl-2-thiazolyl)-2,5-diphenyl -2-*H*-tetrazolium bromide (MTT) were bought from Sigma–Aldrich (Saint. Louis, MO, USA). PrimeScript™ RT reagent kit with gDNA Eraser and SYBR Premix Ex Taq™ II were from TakaraBio (Shiga, Japan). ST2825 and Tak242 were obtained from MedChemExpress (Monmouth Junction, NJ, USA). Rabbit anti-Phospho-NF-*κ*B-p65 (Ser536) (93H1, 1:1000 dilution), rabbit anti-NF-*κ*B-p65 (C22B4) (1:2000), anti-I*κκ*-*β* (1:1000), anti-I*κκ*-*α* (1:1000), anti-Phospho-I*κκ*-*α*/*β*, anti-I*κ*B*α* (1:1000) rabbit anti-p44/42 MAPK (ERK1/2) (137F5, 1:1000) and rabbit anti-phospho-p44/42 MAPK (ERK1/2) (Thr202/Tyr204, 1:1000) were purchased from Cell Signaling Technologies (Beverley, MA, USA). Anti-mouse toll-like receptor 4 (TLR4) (1:1000), antibody rabbit anti-JAK2 (C-20) (SC294, 1:1000), rabbit anti-Stat3 (F-2), (sc8019, 1:1000), rabbit anti-p-Stat3(Ser727) (sc135649 1:1000), goat anti-Akt1 (C-20) (sc-1618 1:1000), mouse anti-JNK (D-2) (sc-7345, 1:1000) and mouse anti-p-JNK (G-7, sc-6254, 1:1000) were from Santa Cruz Biotechnology (Santa Cruz, CA, USA). Rabbit anti-Phospho-JAK2 (PY1007/1008) (#1477-1, 1:1000) was obtained from Epitomics (Burlingame, CA, USA). Rabbit anti-p-Akt1 (PT308) (1:1000) and anti-MyD88 antibody (1:1000) were from Abcam (Cambridge, UK). Goat anti-p38α MAPK (#p3006, 1:1000) was acquired from Abmart (Berekeley Heights, NJ, USA). Pyrrolidine dithiocarbamic acid ammonium salt (PDTC) and Bay 11-7082 (NF-κB inhibitors) were purchased from Sigma–Aldrich and Beyotime Biotechnology (Haimen, China), respectively. Secondary antibodies were anti-rabbit IgG-HRP (1:5000), anti-mouse IgG-HRP (1:5000) and anti-goat IgG-HRP (1:5000) (R&D Systems, Minneapolis, MN, USA). All other reagents were of analytical grade.

### 4.2. Carbohydrate and Protein Contents of Crude and Pure O-Acetylated Glucomannan

The carbohydrate contents of crude and pure *O*-acetylated glucomannan were measured using the anthrone-sulfuric acid reaction [37]. Glucose was used as the standard and the concentration range was 0–72 μg/mL with a coefficient value of 0.994. The concentrations of crude polysaccharide and purified *O*-acetylated glucomannan were 46.1 μg/mL and 40 μg/mL, respectively. The anthrone-sulfuric acid reagent was prepared immediately before use. The protein contents of crude and purified *O*-acetylated glucomannan were measured by the bicinchoninic acid method [38]. The concentrations range was 0–500 ng/mL with a coefficient value of 0.999. The concentrations of crude polysaccharide and purified *O*-acetylated glucomannan were 2 and 2.35 mg/mL, respectively.

### 4.3. Thermal Stability Analysis of O-Acetylated Glucomannan

The thermal stability of *O*-acetylated glucomannan was determined by differential scanning calorimetry (DSC) and size-exclusion chromatography–high-performance liquid chromatography (SEC–HPLC). Samples solutions were prepared at the pH indicated unless specified otherwise (Figure 2). Two sets of identical samples of each polysaccharide were prepared: One set for DSC analysis and the other set for HPLC analysis. The storage temperature of polysaccharides for HPLC analysis was 65 °C. A MicroCal VP-DSC system (Malvern Instruments, Malvern, UK) was used at 25–90 °C with a scan rate of 1 °C/min. Analyses of thermograms were done with Origin™ software (OriginLab, Northampton, MA, USA). All experiments were carried out at a polysaccharide concentration of 2 mg/mL for DSC and HPLC analyses. For SEC-HPLC, 0.1 M NaCl was used as the eluent at a flow rate of 0.5 mL/min and the injection volume was 20 µL; a PL aquagel-OH column (Agilent Technologies, Santa Clara, CA, USA) was employed at 30 °C.

### 4.4. Cell Culture

Human leukemia monocytic (THP-1) cells were purchased from the Kunming Cell Bank of the Chinese Academy of Sciences (Beijing, China). They were cultured in improved RPMI 1640 (HyClone Laboratories, Logan, UT, USA) supplemented with 10% fetal bovine serum (FBS; HyClone Laboratories), 1% penicillin/streptomycin (Invitrogen, Carlsbad, CA, USA) and 50 µM *β*-mercaptoethanol (Amresco, Solon, OH, USA) at 37 °C with a mixture of air and CO_2_ (95% to 5%, *v*/*v*). Cells at passages 3–5 were used for experiments.

### 4.5. MTT Assay

The toxicity of DOP-1-1 against THP-1 cells was tested using a modification of the method described by Carmichael et al. [39]. THP-1 cells (3 × 10^4^/well) were plated with improved complete RPMI 1640 (HyClone Laboratories) supplemented with 0.2 ng/mL PMA into a 96-well plate and incubated for 48 h. DOP-1-1 (25, 50, 100, 150, 300 μg/mL) was added and cells were incubated at 37 °C for 12, 24, 36 and 48 h, respectively. Then, cells were washed twice with phosphate-buffered saline (PBS) and MTT solution (0.5 mg/mL) was added to induce a reaction for 4 h. The purple MTT-formazan crystals were dissolved with 150 µL of dimethyl sulfoxide. Spectrophotometric absorbance at 492 nm was determined immediately using a microplate reader. Percent survival was calculated according to the following formula:

A = A_E_/A_C_ × 100%

where A is the rate of cell growth; A_E_ is the optical density of the experimental group; and A_C_ is the optical density of control group.

### 4.6. Kinetic Evaluation of the Interactions between DOP-1-1 and THP-1 Cells

The surface of a COP-1 chip (compatible polystyrene coated sensor surface) was incubated with 100 µL poly-l-lysine solution (50 µg/mL) for 20 min. Then, the surface was washed with tissue culture-grade water and dried under a sterile hood ≥2 h before the introduction of THP-1 cells. A total of 100,000 THP-1 cells were seeded on the COP-1 surface in 700 μL of medium (improved RPMI 1640 supplemented with 10% FBS, 1% penicillin/streptomycin and 20 ng/mL PMA), and cultured for 48 h at 37 °C under an atmosphere with 5% CO_2_/95% air. Subsequently, the medium was removed, and 700 μL of improved RPMI 1640 without FBS or penicillin/streptomycin was added. After 20 h, one chip was fixed with 3.7% formaldehyde (or not) at 4 °C for 10 min (the unfixed surface was not treated with formaldehyde). The cell coverage on the sensor surface was evaluated by staining the nuclei with Hoechst 33342 and visualized under a fluorescence microscope (Olympus, Tokyo, Japan). Then, the chip was docked into an A200 QCM instrument (Attana, Stockholm, Sweden) and a biosensor experiment measured the interactions between DOP-1-1 and cells.

### 4.7. Western Blotting

THP-1 cells (1 × 10^7^/well) were induced into a mature macrophage-like state as noted above (Section 4.5). After pretreatment with DOP-1-1 (25, 50, 75, 100, 150 μg/mL), the samples of extracted protein were separated by 8% sodium dodecyl sulfate-polyacrylamide gel electrophoresis and then transferred to polyvinylidene difluoride (PVDF) membranes. Cytosol and nuclear proteins were prepared using a Cytosol and Nuclear Extracts kit (Beyotime Biotechnology, ShangHai, China). After blockade with 5% bovine serum albumin (BSA), PVDF membranes were incubated overnight with primary antibodies. After washing three times, a secondary antibody was added to PVDF membranes along with 5% bovine serum albumin for 1 h. *β*-tubulin was used as an internal control.

The primary antibodies used were rabbit anti-Phospho-NF-*κ*B-p65, rabbit anti-NF-*κ*B-p65 anti-I*κκ*-*β*, anti-I*κκ*-*α*, anti-Phospho-I*κκ*-*α*/*β*, anti-I*κ*B*α*, rabbit anti-JAK2, rabbit anti-Phospho-JAK2, rabbit anti-Stat3, rabbit anti-p-Stat3, goat anti-Akt1, rabbit anti-p-Akt1, rabbit anti-p44/42 MAPK (ERK1/2), rabbit anti-phospho-p44/42 MAPK(ERK1/2), goat anti-p38(MAPK), anti-phospho-p38(MAPK), mouse anti-JNK, and mouse anti-p-JNK. The secondary antibodies used were anti-rabbit IgG-HRP, anti-mouse IgG-HRP, and anti-goat IgG-HRP.

For visualization of the protein bands, a detection system of enhanced chemiluminescent, colorimetric and UV fluorescent gels and blots (FluorChem E system; ProteinSimple, Santa Clara, CA, USA) was applied.

### 4.8. Immunofluorescent Staining

THP-1 cells (1 × 10^6^) were plated onto a six-well plate in a total volume of 2 mL culture medium containing 0.2 ng/mL PMA. After attachment, cells were cultured in the medium with 5% FBS for 20 h. Then, cells were fixed in paraformaldehyde for 20 min at room temperature and washed gently thrice with PBS. Afterwards, cells were blocked with 1% BSA for 30 min and incubated with anti-p-NF-*κ*B/p65 Ser536 antibody in 0.1% BSA at 4 °C overnight. Subsequently, cells were rinsed three times with PBS at room temperature and labelled with secondary antibodies with Alexa Fluor 488 (donkey anti-rabbit, Life Technologies, Carlsbad, CA, USA) for 1 h in the dark. The expression of NF-*κ*B was photographed using a confocal microscope.

### 4.9. Total Rna Extraction and Real-Time Reverse Transcription-Polymerase Chain Reaction (Rt-Pcr)

Total RNA was isolated using TRIzol^®^ (TakaraBio) following the manufacturer’s instructions. The concentration and purity of RNA were determined by measuring absorbance at 260 and 280 nm using a NanoDrop spectrophotometer (Beckman Coulter, Brea, CA, USA). DNA Eraser consisted of 2 μg of total RNA, 4 μL of 5× gDNA Eraser buffer, 2 μL of gDNA Eraser, complemented with RNase Free water to a total volume of 20 μL. The procedure was carried out for 2 min at 42 °C. RT (reverse transcription) consisted of 20 μL of the reaction solution stated above, 2 μL of RT Primer Mix, 8 μL of 5× PrimeScript Buffer 2, and 2 μL of PrimeSCript RT Enzyme Mix I complemented with PCR water to a total volume of 40 μL. The reverse transcription reactions were carried out at 37 °C for 15 min and 85 °C for 5 s. Target mRNA was quantified by real-time RT-PCR (TaqMan^®^) using a 7900 HT Fast Real-Time PCR system (Applied Biosystems, Foster City, CA, USA). Real-time RT-PCR was carried out in a 384-well plate (in triplicate) using SYBR Green Real Time PCR Master Mix according to the manufacturer’s instructions. TaqMan threshold cycle number (Ct) was normalized using the 2^∆∆Ct^ method:

∆∆Ct = (Ct_Target_ − Ct_Actin_) treatment − (Ct_Target_ − Ct_Actin_) control

The primers used in this study are listed in Table 2. After the PCR, the amplicon melting curve was checked for PCR specificity.

### 4.10. Statistical Analysis

The results are expressed as the means ± standard deviations (SD) of data obtained from triplicate experiments. Statistical analyses were performed using a one-way Analysis of Variance (ANOVA) with Prism6 software (GraphPad, San Diego, CA, USA). *p* < 0.05, *p* < 0.01, or *p* < 0.001 were considered as statistically significant (* *p* < 0.05, ** *p* < 0.01, or *** *p* < 0.001).

## 5. Conclusions

The *O*-acetylated glucomannan DOP-1-1 is present in the steady state in low-pH solutions. DOP-1-1 may induce an immune response through NF-*к*B mediated by a TLR4 signaling pathway. CCL4 and IP10 may be the novel targets of the immune response stimulated by *O*-acetylated glucomannan.

## Figures and Tables

**Table 1:** Carbohydrate and Protein Contents of Crude polysaccharide and purified *O*-acetylated glucomannan.

Sample	Total Carbohydrate (% *w*/*w*)	Protein (% *w*/*w*)
Crude polysaccharide (DOP)	76	0.002
*O*-acetylated glucomannan (DOP-1-1)	98.9 ± 0.74	Not Detected

**Table 2:** Sequences of the primers and probes used in real-time PCR assays.

Gene Name	Forward Primer	Reverse Primer
β-actin	AATCTGGCACCACACCTTCTAC	ATAGCACAGCCTGGATAGCAAC
iNOS	TACTCCACCAACAATGGCAA	ATAGCGGATGAGCTGAGCAT
MCP1	AAGCAGAA GTGGGTTCAGGA	TAAAACAGGGTGTCTGGGGA
BIRC2(cIAP1)	GAATCTGGTTTCAGCTAGTCTGG	GGTGGGAGATAATGAATGTGCAA
IFNβ	GTCTCCTCCAAATTGCTCTC	ACAGGAGCTTCTGACACTGA
BCL-X-Long	CATGGCAGCAGTAAAGCAAG	TGCTGCATTGTTCCCATAGA
BIRC3(cIAP2)	AAGCTACCTCTCAGCCTACTTT	CCACTGTTTTCTGTACCCGGA
Bcl-2	GAGGATTGTGGCCTCTTTG	ACAGTTCCACAAAGGCATCC
c-FLIP	AATTCAAGGCTCAGAAGCGA	GGCAGAAACTCTGCTGTTCC
gadd45β	ACAGTGGGGGTGTACGAGTC	TTGATGTCGTTGTCACAGCA
TRAF1	AGAACCCGAGGAATGGCGA	TGAAGGAGCAGCCGACACC
TRAF2	AGGTCTGCCCCAAGTTCCC	GCTGTTTCTCACCCTCTACCGT
A1/Bf1-1	CAGGCTGGCTCAGGACTATC	TGTTCTGGCAGTGTCTACGG
IL8	GTCCTTGTTCCACTGTGCCT	GCTTCCACATGTCCTCACAA
IL-1α	TGGCTCATTTTCCCTCAAAAGTTG	AGAAATCGTGAAATCCGAAGTCAAG
IL-1β	ACGAATCTCCGACCACCACT	CCATGGCCACAACAACTGAC
TNF-α	CCCCAGGGACCTCTCTCTAATC	GGTTTGCTACAACATGGGCTAC A
A20	TCCTCAGGCTTTGTATTTGAGC	TGTGTATCGGTGCATGGTTTTA
IL-6	GTGTGAAAGCAGCAAAGAG	CTCCAAAAGACCAGTGATG
IL-12	TGGAGTGCCAGGAGGACAGT	TCTTGGGTGGGTCAGGTTTG
COX2	TTCTCCTTGAAAGGACTTATGGGTAA	AGAACTTGCATTGATGGTGACTGTTT
MIP2(CXCL2)	TTCACAGTGTGTGGTCAACATTT	TCTGCTCTAACACAGAGGGAAAC
VCAM-1	TGGGCTGTGAATCCCCATCT	GGGTCAGCGCGTGGAATTGGTC
uPA	ATCTGCCTGCCCTCGATGTATAA	TTTCAGCTGCTCCGGATAGAGATAG
BLC	ACTCTGCTAATGAGCCTGGAC	CCTTGGACTGGAGAGAGGCT
ELC(CCL19)	CCATCCCTGGGTACATCGTG	GCAGTCTCTGGATGATGCGT
SLC	GTCTCCCAGGGAGCATGAGA	GGGAGCCGTATCAGGTCCA
SDF-1α	ACTAAAACCTTGTGAGAGATGA	GGGTCTAAATGCTGCAAACCT
BAFF	GGCAGGTACTACGACCATCTC	TGGGCCTTTTCTCACAGAAGT
ICAM1	CCCATGAAACCGAACACAC	ACTCTGTTCAGTGTGGCACC
MIP-1α	ATGCAGGTCTCCACTGCTG	TCAGGCACTCAGCTCCAG
CXCL1	AGGGAATTCACCCCAAGAAC	CACCAGTGAGCTTCCTCCTC
CCL2	CTTCTGTGCCTGCTGCTCAT	CGGAGTTTGGGTTTGCTTGTC
IP10	GGGAGCAAAATCGATGCAGTGCT	GCAGCCTCTGTGTGGTCCATCC
IFNα	CCTGATGAAGGAGGACTCCATT	AAAAAGGTGAGCTGGCATACG
CCL4(MIP1β)	GCTAGTAGCTGCCTTCTGCTCTCC	CAGTTCCAGCTGATACACG TACTCC
CCL5	CTGCTGCTTTGCCTACATTGC	GTTCAGGTTCAAGGACTCTCCATC
CXCL10	AAGCAGTTAGCAAGGAAAGGTC	TTGAAGCAGGGTCAGAACATC
IL-2	GAACTAAAGGGATCTGAAACAACATTC	TGTTGAGATGATGCTTTGACAAAA
IL-4	TGCTTCCCCCTCTGTTCTTCCT	GGCAGCGAGTGTCCTTCTCATG
IL-5	AGCCATGAGGATGCTTCTGC	AAGCAGTGCCAAGGTCTCTT
IL-10	GCCTAACATGCTTCGAGATC	CTCATGGCTTTGTAGATGCC
CDK4	AGTTCGTGAGGTGGCTTTA	GGGTGCCTTGTCCAGATA
GMCSF	TCAGGATGGTCATCTTGGAG	TCTTCTGCCATGCCTGTATC
IκBα	CTCCGAGACTTTCGAGGAAATAC	GCCATTGTAGTTGGTAGCCTTCA
NF-κB(p50)	CCTGGATGACTCTTGGGAAA	TCAGCCAGCTGTTTCATGTC
NF-κB(p52)	GAACAGCCTTGCATCTAGCC	TTTTCAGCAT GGATGTCAGC
p65(RelA)	TCTGCTTCCAGGTGACAGTG	GCCAGAGTTTCGGTTCACTC
c-Rel	CGAACCCAATTTATGACAAC	TTTTGTTTCTTTGCTTTATTGC
RelB	CTGCTTCCAGGCCTCATATC	CGCAGCTCTGATGTGTTTGT
TLR4	AGAAGCAGTGAGGATGATGCC	TTCTGTGTGGTTTAGGGCCA
MYD88	GAAGCCTAGAGGCCATTCTG	GGCTTGTGATCTCAGGTGAA
TRAF6	CACGTGGATACCAACTGCTC	TGTGTGCATCTCCTGTCTTG
